# Altered Long- and Short-Range Functional Connectivity Density in Patients With Thyroid-Associated Ophthalmopathy: A Resting-State fMRI Study

**DOI:** 10.3389/fneur.2022.902912

**Published:** 2022-06-23

**Authors:** Wen-Hao Jiang, Huan-Huan Chen, Wen Chen, Qian Wu, Lu Chen, Jiang Zhou, Xiao-Quan Xu, Hao Hu, Fei-Yun Wu

**Affiliations:** ^1^Department of Radiology, The First Affiliated Hospital of Nanjing Medical University, Nanjing, China; ^2^Department of Endocrinology, The First Affiliated Hospital of Nanjing Medical University, Nanjing, China

**Keywords:** thyroid-associated ophthalmopathy, resting-state functional magnetic resonance imaging, functional connectivity density, prefrontal cortex, parietal lobe

## Abstract

**Background and Purpose:**

Although previous neuroimaging studies have demonstrated emotion- and psychology-associated brain abnormalities in patients with thyroid-associated ophthalmopathy (TAO), the changes of brain functional connectivity in TAO were seldom focused. We aimed to investigate interregional and intraregional functional interactions in patients with TAO by using resting-state functional MRI (rs-fMRI) with long- and short-range functional connectivity density (FCD) analysis.

**Methods:**

Thirty patients with TAO and 30 well-matched healthy controls (HCs) were recruited in our study. Long- and short-range FCD values were calculated and compared between the two groups. Correlations between long- and short-range FCD values and clinical indicators were analyzed.

**Results:**

Compared with HCs, patients with showed both increased long- and short-range FCDs in the left middle frontal gyrus (MFG), orbital part of superior frontal gyrus (ORBsup), and dorsolateral part of superior frontal gyrus (SFGdor); meanwhile, both decreased long- and short-range FCDs in bilateral postcentral gyrus (PoCG), left superior parietal gyrus (SPG), and inferior parietal (IPL). In addition, patients with TAO showed increased short-range FCD in the right SFGdor, bilateral medial part of superior frontal gyrus (SFGmed), left orbital part of middle frontal gyrus (ORBmid), and orbital part of inferior frontal gyrus (ORBinf), as well as decreased short-range FCD in the right supplementary motor area (SMA) and the left paracentral lobule (PCL) than HCs. Moreover, the short-range value in the left SFGdor showed a negative correlation with Montreal Cognitive Assessment (MoCA) score (*r* = −0.501, *p* = 0.005).

**Conclusion:**

Our findings complemented the functional neural mechanism of TAO, and provided potential neuroimaging markers for assessing the psychiatric, visual, and emotional disturbances in patients with TAO.

## Introduction

Thyroid-associated ophthalmopathy (TAO) is an autoimmune orbital disorder which mainly targets adults of 30–50 years old (1). Although the pathogenesis of TAO is not yet entirely clear, the antigen shared by the thyroid gland and the orbit, primarily thyroid stimulating hormone receptor, may mediate the autoimmune response (2). Despite that TAO patients commonly complain about the ocular symptoms (3), the additional psychological and psychiatric disturbances can also markedly reduce the patients' quality of life (4). Specifically, cognitive function such as performing daily work and maintaining social relationships (5), and emotion regulation ability such as relieving depression and anxiety (6) have been reported to be impaired. A prior cohort study even showed a higher suicidal tendency for TAO patients (7). These evidences indicated that TAO might also be a neuropsychiatric disease, rather than merely orbital involvement (8).

Recently, increasing researchers tried to use MRI techniques to explore the brain alterations in TAO patients. Structural neuroimaging studies have revealed both morphological and microstructural abnormalities corresponding to visual and cognitive deficits, and destruction of structural brain network connectome in TAO patients (9, 10). Meanwhile, several case-control studies have used functional neuroimaging methods, including amplitude of low-frequency fluctuation, regional homogeneity, and degree centrality, to evaluate the brain functional abnormality in TAO patients. They reported that TAO patients exhibited aberrant brain function involving default mode, visual, executive control, sensorimotor, and attention networks (11–13). However, the above-mentioned studies were only preliminary explorations using basic methods and were mainly concerned with functional segregation, which ignored the alterations of brain functional integration in TAO patients. Functional connectivity belongs to a useful measurement reflecting changes of brain functional integration (14), while it was seldom focused in studies on TAO. Until now, only one study reported the interhemispheric functional connectivity alterations of TAO using voxel-mirrored homotopic connectivity (15). However, it ignored the interregional and intraregional functional interactions of the brain. Inter-/intra- regional functional connectivity is suitable for reflecting widespread alterations in functional integration when deficits occur, and provides complementary information for a more targeted analysis (16). Besides, compared with other functional integration methods such as hypothesis-driven resting-state functional connectivity analysis, it can objectively and comprehensively reflect the abnormalities of functional connectivity within the whole-brain network (17).

Functional connectivity density (FCD) analysis, a data-driven and graph-based approach, has been widely used in resting-state functional MRI (rs-fMRI) studies. It is mainly used to quantify global and local spontaneous neural activity. FCD is divided into long-range and short-range FCDs, reflecting interregional and intraregional functional interactions, respectively. Previously, the method has already been applied in several ocular diseases, such as glaucoma (17), neuromyelitis optica (16), corneal ulcer (18), and eye pain (19), to explore changes in global and local functional connectivity of the brain. Hence, we hypothesized that the underlying alterations of interregional and intraregional functional interactions in the TAO cohort could also be visualized by long- and short-range FCDs, which may be a supplement to the functional neural mechanism of TAO.

Therefore, the purposes of our study were to (1) investigate underlying global and local spontaneous neural activity alterations using rs-fMRI with FCD analysis, and (2) relate the long- and short-range FCD values to the clinical parameters and scales in patients with TAO.

## Materials and Methods

### Subjects

Consecutively, 30 patients with TAO (18 females and 12 males, mean age 43.27 ± 14.20 years) were recruited from the department of endocrinology in our hospital. Concurrently, 30 healthy volunteers (18 females and 12 males, mean age 43.47 ± 13.62 years) were recruited from the local community, who were matched for age and gender with the patient group. All patients enrolled were in euthyroid status for at least 3 months (Reference ranges: serum free triiodothyronine, 3.10–6.80 pmol/L; free thyroxine, 12.00–22.00 pmol/L; thyroid-stimulating hormone, 0.270–4.200 mIU/L), in order to avoid the potential interference from thyroid hormone to the inner neural pattern of TAO. The exclusion criteria which were applied to all subjects were as follows: (1) local eye disorders caused by other reasons (amblyopia, strabismus, glaucoma, cataracts, etc.); (2) history of eye surgery; (3) history of neurological and psychiatric diseases (head injury, bipolar disorder, schizophrenia, etc.); (4) alcohol or drug addiction; (5) contraindications to MRI examination (implanted metal devices or cardiac pacemaker); (6) inadequate image quality. Comorbid depression and (or) anxiety symptoms were not regarded as exclusion criteria if TAO was the primary clinical diagnosis.

### Clinical Assessment

Patients with TAO were clinically diagnosed by two experienced endocrinologists (with 17 and 35 years of experience in TAO assessment, respectively). The duration of TAO disease was defined from the onset of associated ophthalmic symptoms to the date of MRI examination. The scoring of disease activity was performed according to the modified 7-point clinical activity score (CAS) (20). Besides, the visual acuity and degree of exophthalmos were measured for each participant, and the values of the worse eye were recorded.

Cognitive and psychometric assessments were conducted for each subject before the MRI scan. Hamilton Depression Rating Scale (HDRS) and Hamilton Anxiety Rating Scale (HARS) were used to assess the depressive symptoms and anxiety symptoms, respectively. Cognitive function was measured using Montreal Cognitive Assessment (MoCA). In addition, TAO-specific quality of life (QoL) questionnaire containing visual functioning and appearance was applied to each patient before the MRI scan (21).

### MRI Data Acquisition

A 3.0-T MRI system (MAGNETOM Skyra; Siemens Healthcare, Erlangen, Germany) equipped with a 20-channel head coil was applied to acquire rs-fMRI data. Foam pads and earplugs were used to reduce head movement and scanning noise. Each participant was instructed to remain still in the supine position, with eyes closed and awake during the scan. An axial gradient echo planar imaging sequence was applied to acquire functional images with the following parameters: number of slices = 35, repetition time (TR) = 2,000 ms, echo time (TE) = 30 ms, flip angle = 90°, field of view (FOV) = 240 × 240 mm^2^, matrix = 64 × 64, thickness = 4.0 mm, and voxel size = 3.75 × 3.75 × 4 mm^3^. High-resolution sagittal structural T1-weighted images were also obtained with the following parameters: number of slices = 176, TR = 1,900 ms, TE = 2.45 ms, flip angle = 9°, FOV = 256 × 256 mm^2^, matrix = 256 × 256, thickness = 1.0 mm, and voxel size = 1 × 1 × 1 mm^3^. The total scanning duration was 12 min and 26 s.

### fMRI Data Preprocessing

RESTplus V1.21 (http://www.restfmri.net/forum/RESTplusV1.2) (22) was used to preprocess the rsfMRI data, which is based on SPM12 (http://www.fil.ion.ucl.ac.uk/spm/software/spm12) (23). The main processes were as follows: (1) the first ten volumes of each data were removed to reduce the influence of machine instability and subject adaptation to the environment; (2) slice timing correction was performed on the data of the remaining 230 time points; (3) realignment for head motion correction; (4) the realigned functional images were normalized to the Montreal Neurological Institute template by using T1 image unified segmentation; (5) and then the functional images were resampled to 3 × 3 × 3 mm^3^ voxel; (6) detrending was conducted to remove linear trend; (7) the Friston 6-parameter model, cerebrospinal fluid, and white matter were regressed as covariates in MNI space; (8) bandpass filtering (based on frequency 0.01–0.08 Hz) was performed to reduce the effects of low frequency drift and high frequency noise. The whole dataset of this subject would be abandoned, if the maximum value of the head translation (rotation) movement was more than 2.0 mm (2.0°).

### Resting-State FCD Calculation

Long- and short-range FCD calculations were performed using a Graph-theoretical Network Analysis Toolkit (GRETNA v1.2.1) (http://www.nitrc.org/projects/gretna/), which is based on the theory put forward by Tomasi and Volkow (24). In binary networks, FCD is defined as the number of functional connections for a given voxel, which is determined by setting a threshold to derive the Pearson's correlation coefficient of time-varying signals between one voxel and any other voxel. The correlation coefficient threshold (Tc) was set to 0.25 to keep a balance between false positive rate and sensitivity. In addition, if a correlation factor (*r*) > 0.25, functional connectivity can be considered to exist (25). According to the study of He et al. (26), we classified functional connectivity into long- and short-range by a given anatomical distance of 75 mm. Short-range FCD was defined as the number of voxels that have functional connections to any voxel within the local area, while long-range FCD referred to the number of voxels that have functional connections to a voxel outside the local functional mass. Finally, Z-transformation was performed for the values of long- and short-range FCD maps to improve the normality of the distribution. Then, the Z-score transformed FCD data were spatially smoothed with a Gaussian kernel of 6 × 6 × 6 mm^3^ depending on full width at half maximum (FWHM) to eliminate artifacts and increase the SNR.

### Statistical Analyses

All clinical and demographic variables in our study were statistically analyzed using SPSS software (SPSS version 25.0, Inc., Chicago, IL, United States). For normally distributed variables, Student *t-*tests were employed to compare the differences between TAO and HC groups. For the non-parametric variables, Mann-Whitney *U-*tests were applied to analyze the differences between the two groups. Chi-square tests were used to analyze the differences of categorical data between the two groups. Statistical significance was set as *p* < 0.05, with all tests being two-tailed.

For the long- and short-range FCD values, statistical analyses were calculated by using SPM12. Independent two-sample *t*-tests were used to compare the differences of FCD values between TAO and HC groups with a gray-matter mask. Statistical significance was based on the current reporting guideline (27) to reduce the false positive rate, with a corrected threshold of voxel *p* < 0.001, cluster *p* < 0.05. With the peak voxels of significant brain regions in two-sample tests as spherical centers, spherical regions of interest (ROIs) (radius = 6 mm) of altered long- and short-range FCDs were created and extracted for each patient with TAO. Then, Pearson's and Spearman's correlation analyses were conducted to analyze the relationships between clinical data and FCD values within the group with TAO, with a statistical threshold of *p* < 0.05.

## Results

### Demographic and Clinical Data

Demographic and clinical data of TAO and HC groups are presented in Table 1. Two groups were well matched for age (*p* = 0.956) and gender (*p* > 0.999). Compared to HCs, patients with TAO showed significantly reduced visual acuity (*p* < 0.001). Besides, patients with TAO scored higher in HARS (*p* < 0.001) and HDRS (*p* < 0.001), while lower in MoCA (*p* < 0.001) than HCs.

**Table 1:** Demographic and clinical characteristics of the group with TAO and HCs.

**Items**	**TAO group (*n* = 30)**	**HCs (*n* = 30)**	***p*-value**
Age (years)	43.27 ± 14.20	43.47 ± 13.62	0.956
Gender (female/male)	18/12	18/12	>0.999
Disease duration (months)	10.03 ± 11.53	-	
CAS	3.17 ± 1.05	-	
FT3 (pmol/L)	4.67 ± 0.76	4.92 ± 0.96	0.337
FT4 (pmol/L)	16.39 ± 2.83	16.80 ± 2.09	0.529
TSH (mIU/L)	1.77 ± 1.83	1.63 ± 1.13	0.679
Visual acuity	0.76 ± 0.15	0.97 ± 0.15	<0.001
Exophthalmos (mm)	20.20 ± 3.57	-	
**QoL scores**			
Visual functioning	56.98 ± 31.36	-	
Appearance	65.60 ± 21.27	-	
Total score of HARS	17.13 ± 7.95	2.50 ± 1.20	<0.001
Total score of HDRS	16.53 ± 6.95	2.33 ± 1.67	<0.001
Total score of MoCA	25.87 ± 3.35	28.77 ± 1.10	<0.001

*Data were presented as mean ± SD*.

*CAS, clinical activity score; FT3, free triiodothyronine; FT4, free thyroxine; TSH, thyroid-stimulating hormone; QoL, quality of life; HDRS, Hamilton Depression Rating Scale; HARS, Hamilton Anxiety Rating Scale; MoCA, Montreal Cognitive Assessment; TAO, thyroid-associated ophthalmopathy; HCs, healthy controls*.

### FCD Analysis

**Figure 1:**
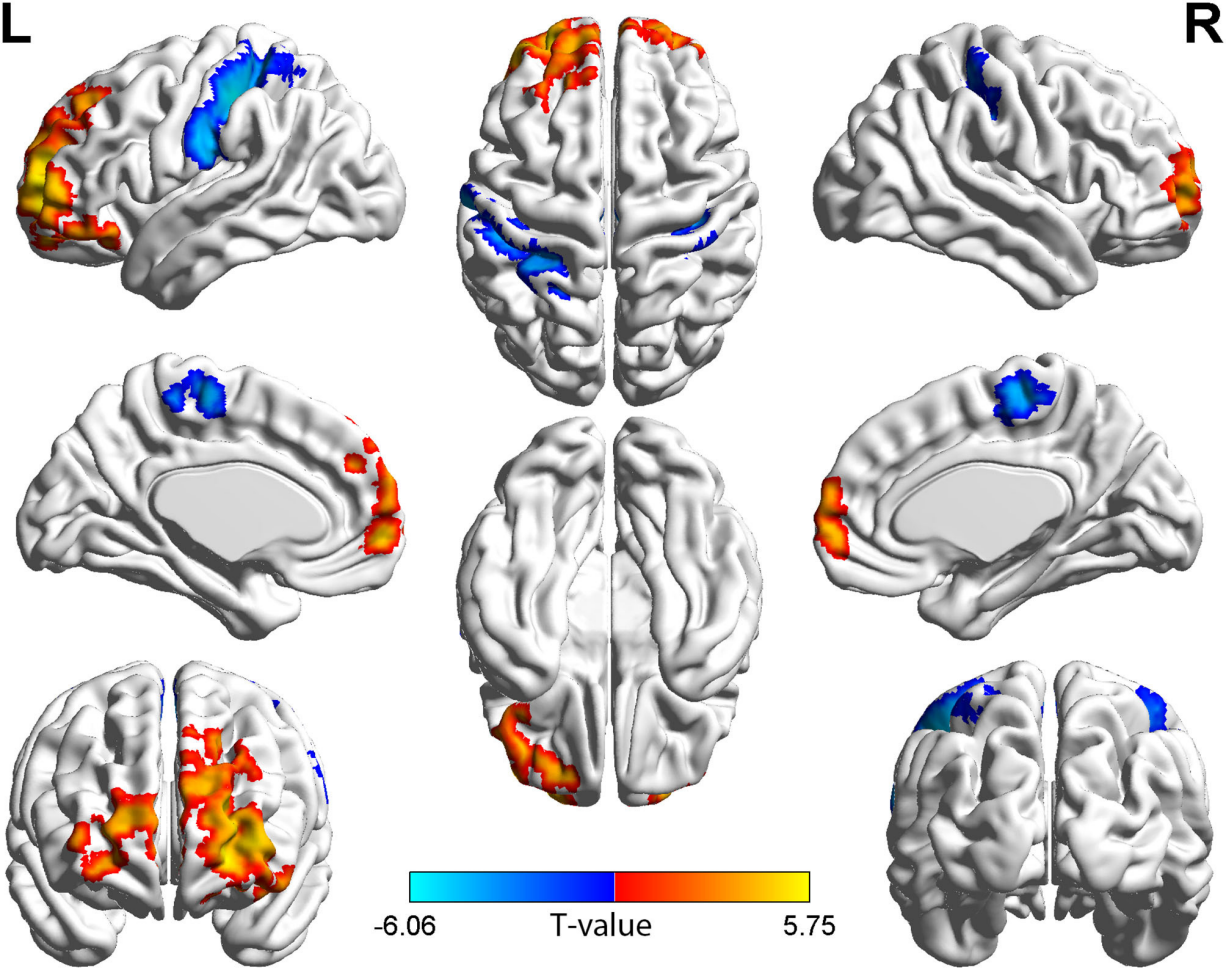
Brain regions with short-range FCD difference between the group with TAO and HCs. Compared with HCs, the group with TAO showed significantly increased short-range FCD in the bilateral SFGdor and SFGmed, left MFG, ORBmid, ORBinf, and ORBsup, and significantly decreased short-range FCD in the bilateral PoCG, left SPG, IPL, PCL, and right SMA (voxel *p* < 0.001, cluster *p* < 0.05, cluster-level FWE corrected, cluster size ≥ 109 voxels). The color bar indicates the *T-*value from two-sample *t-*tests between the group with TAO and HCs, the warm color denotes relatively higher short-range FCD in the group with TAO, and the cold color denotes relatively lower short-range FCD in the group with TAO. FCD, Functional connectivity density; SFGdor, dorsolateral part of superior frontal gyrus; SFGmed, medial part of superior frontal gyrus; MFG, middle frontal gyrus; ORBmid, orbital part of middle frontal gyrus; ORBinf, orbital part of Inferior frontal gyrus; ORBsup, orbital part of superior frontal gyrus; PoCG, postcentral gyrus; SPG, Superior parietal gyrus; IPL, Inferior parietal; PCL, paracentral lobule; SMA, supplementary motor area; FWE, family-wise error.

**Figure 2:**
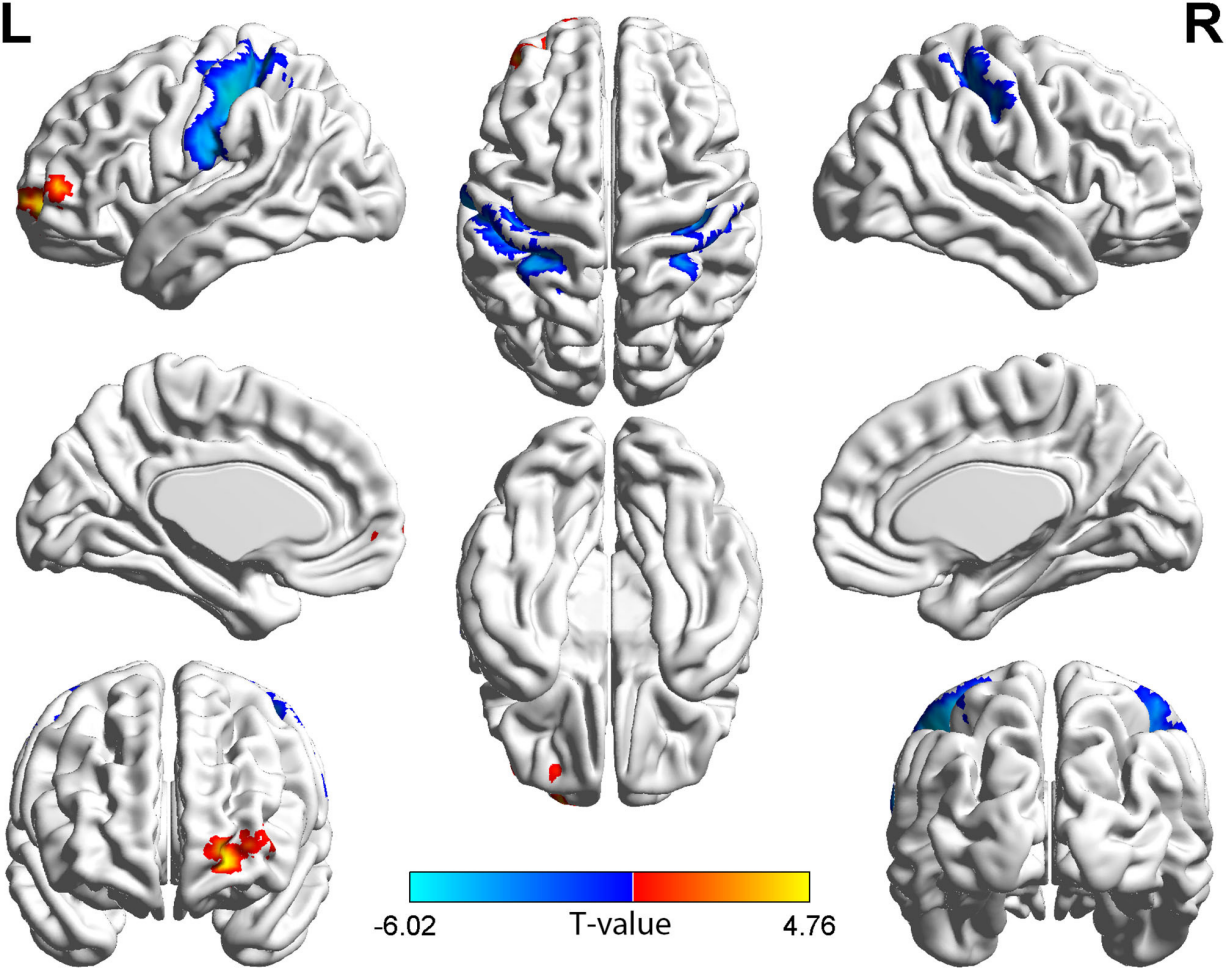
Brain regions with long-range FCD difference between the group with TAO and HCs. Compared with HCs, the group with TAO showed significantly increased long-range FCD in the left MFG, ORBsup, and SFGdor, and significantly decreased long-range FCD in the bilateral PoCG, left SPG, and IPL (voxel *p* < 0.001, cluster *p* < 0.05, cluster-level FWE corrected, cluster size ≥ 114 voxels). The color bar indicates the T value from two-sample *t-*tests between the group with TAO and HCs, the warm color denotes relatively higher long-range FCD in the group with TAO, and the cold color denotes relatively lower long-range FCD in the group with TAO. FCD, Functional connectivity density; MFG, middle frontal gyrus; ORBsup, orbital part of superior frontal gyrus; SFGdor, dorsolateral part of superior frontal gyrus; PoCG, Postcentral gyrus; SPG, Superior parietal gyrus; IPL, Inferior parietal; FWE, family-wise error.

In the group with TAO, the region of the left middle frontal gyrus (MFG), orbital part of superior frontal gyrus (ORBsup), and dorsolateral part of superior frontal gyrus (SFGdor) showed both significantly higher long- and short-range FCD values than in the HC group (*p* < 0.05, cluster-level FWE corrected). Meanwhile, in the group with TAO, the region of bilateral postcentral gyrus (PoCG), left superior parietal gyrus (SPG), and inferior parietal (IPL) showed both significantly lower long- and short-range FCD values than in the HC group (*p* < 0.05, cluster-level FWE corrected). In addition, only significantly increased short-range FCD in the right SFGdor, bilateral medial part of superior frontal gyrus (SFGmed), left orbital part of middle frontal gyrus (ORBmid), and orbital part of inferior frontal gyrus (ORBinf) (*p* < 0.05, cluster-level FWE corrected), as well as decreased short-range FCD in the right supplementary motor area (SMA) and the left paracentral lobule (PCL), were observed in the group with TAO as compared to the HCs (*p* < 0.05, cluster-level FWE corrected). The detailed information for all brain regions with significant long- and short-range FCDs between the two groups are presented in Table 2 and Figures 1, 2.

**Table 2:** Brain areas with significantly different short- and long-range FCD values between groups (*P* < 0.05, cluster-level FWE-corrected).

		**MNI coordinates**				
**Brain regions/conditions**	**BA**	**X**	**Y**	**Z**	**Cluster size (number of voxels)**	***t*-value**
**Short-range FCD:**						
**TAO group** **>** **HCs**						
SFGdor.L/R,	9/10/11/46/47	−24	57	0	1,140	5.746
MFG.L,						
SFGmed.L/R,						
ORBmid.L,						
ORBinf.L,						
ORBsup.L						
**TAO group** **<** **HCs**						
PoCG.L, SPG.L, IPL.L	2/3	−45	−27	48	482	−6.057
PoCG.R	3	39	−33	57	130	−4.400
SMA.R, PCL.L	4/6	−3	−21	60	109	−4.466
**Long-range FCD:**						
**TAO group** **>** **HCs**						
MFG.L, ORBsup.L, SFGdor.L	10/11/46/47	−24	57	−3	114	4.759
**TAO group** **<** **HCs**						
PoCG.L, SPG.L, IPL.L	2/3	−45	−27	48	482	−6.057
PoCG.R	3	39	−30	54	234	−4.589

*FCD, Functional connectivity density; FWE, family-wise error; BA, Brodmann's areas; MNI, Montreal Neurologic Institute; TAO, thyroid associated ophthalmopathy; HCs, healthy controls; R, right; L, left; SFGdor, dorsolateral part of superior frontal gyrus; MFG, middle frontal gyrus; SFGmed, medial part of superior frontal gyrus; ORBmid, orbital part of middle frontal gyrus; ORBinf, orbital part of Inferior frontal gyrus; ORBsup, orbital part of superior frontal gyrus; PoCG, postcentral gyrus; SPG, superior parietal gyrus; IPL, inferior parietal; SMA, supplementary motor area; PCL, paracentral lobule*.

### Correlation Analysis

**Figure 3:**
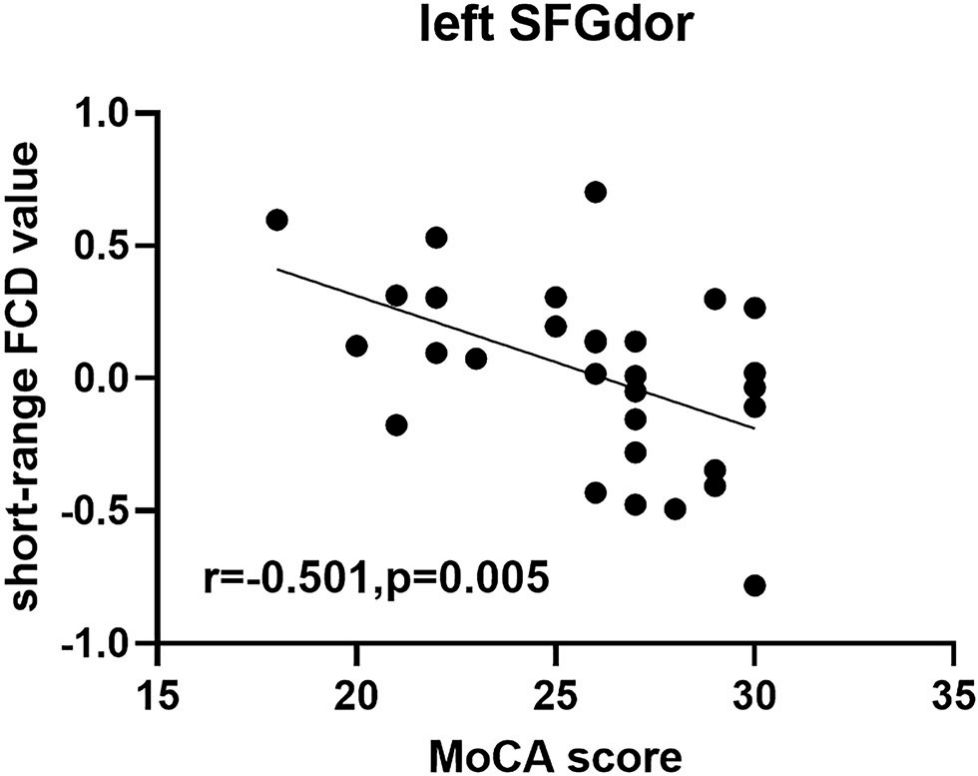
Scatter diagram showing the significant correlation between clinical index and short-range FCD value in the SFGdor in the group with TAO. Short-range FCD value was negatively correlated with the MoCA score (*r* = −0.501, *p* = 0.005). FCD, Functional connectivity density; SFGdor, dorsolateral part of superior frontal gyrus.

In the group with TAO, short-range FCD value in the left SFGdor was negatively correlated with the MoCA score (*r* = −0.501, *p* = 0.005) (Figure 3). No significant correlation was found between the FCD values and any other clinical measures (*p* > 0.05).

## Discussion

In this study, the group with TAO demonstrated increased short-range FCD values in the bilateral SFGdor and SFGmed, and left MFG, ORBmid, ORBinf and ORBsup, as well as increased long-range FCD values in the left MFG, ORBsup, and SFGdor compared with the HC group. The involved brain regions are all important components of the prefrontal cortex, which is generally considered to be associated with cognitive function (28, 29). Previous rsfMRI studies regarding aging associated disorders, including Alzheimer's disease, Parkinsonism, frontotemporal dementia, and post-ischemic stroke, have reported aberrant functional connectivity in the prefrontal cortex (30, 31). Aging associated disorders are usually accompanied by cognitive dysfunction, and the prefrontal cortex is thought to perform top-down control by connecting other brain regions to enable complex cognitive processes (32). Therefore, the altered long- and short-range FCDs of the patient group in this study may also indicate the cognitive impairment related with TAO. Besides, as a part of the cognition functions, social cognition requires the integration of multiple behaviors, including reward-seeking, salience, motivation, and knowledge of self and others, which are also conducted by the prefrontal cortex (33). Patients with TAO were reported to occasionally present with social barriers due to altered appearance or other complicated factors (34), which may also contribute to the abnormal functional connectivity of the prefrontal cortex.

However, interestingly, both the long- and short-range FCDs of prefrontal cortex were increased in the group with TAO, but not the expected decreased values. Coincidentally, one study using the method of regional homogeneity also reported increased value of the prefrontal cortex in TAO (13). Hence, as reference to prior knowledge, the currently increased FCD values might be regarded as a compensatory mechanism in the brain owing to cognitive impairment (16, 35). The negative correlation between the FCD value and MoCA score could further help verify the above deduction, and suggest that the compensatory changes in the prefrontal cortex may progress with cognitive decline.

Another significant finding of our study was the reduction of both long- and short-range FCDs in bilateral PoCG and left SPG and IPL in the group with TAO. The PoCG and SPG participate in the formation of dorsal visual pathway, which is responsible for processing spatial information (17). In recent years, several rs-fMRI studies focusing on other orbital diseases (e.g., glaucoma, exotropia, and blindness) have demonstrated decreased functional connectivity in the PoCG (17, 36, 37). As for the IPL, Nardella et al. (38) suggested that this region was involved in encoding visual temporal resolution by changing the critical flicker frequency threshold. Taken together, the currently reduced FCD values in these regions could also correspond to the visual impairment in TAO. In addition, PoCG is also thought to be involved in the encoding of pain and touch due to its location in the primary sensory cortex, which is essential for sensory functions (39). Considering that the ocular discomforts such as conjunctival hyperemia and eyelid edema commonly occur in TAO patients, we speculated that these somatosensory symptoms may partially relate to the altered functional connectivity in the PoCG.

In addition, the group with TAO showed a decreased short-range FCD in the right SMA and left PCL, compared with the HCs. SMA is located anterior to the primary motor cortex, playing an important role in digital cognition, sports, space and time processing, language and music processing, and working memory (40). Previous studies suggested that SMA is a key hub for depressive episodes in Parkinson's disease (41), and significantly decreased glucose metabolism in SMA is one of the characteristics of patients with refractory depression (42). Besides that, a systematic review reported that transcranial direct current stimulation of the SMA can relieve anxiety symptoms of patients with obsessive-compulsive disorder (43). PCL has bidirectional connections with precuneus (44), which is an important component of default mode network associated with mood disorder (45). Lots of research have shown structural and functional alterations in the PCL of depression and depression-related disorders (46, 47). Therefore, combing the elevated HARS and HDRS scores in the patient group, the reduction of local functional connectivity in SMA and PCL may also be associated with the emotional disturbance in patients with TAO.

Summarizing all the results, our study had the following interesting findings: First, there were many overlap results observed between long- and short-range FCDs. Similar results have been reported in previous rs-fMRI researches using FCD analysis regarding other diseases (16, 19). These results might be due to the fact that long- and short-range FCDs are not completely independent, but interrelated (48). Deco et al. (49) has shown that the functional property of local (short-range) connectivity can influence remote (long-range) connectivity through feedback inhibition. Second, short-range FCD results were more abundant than long-range FCD results between TAO and HC groups. Short-range FCD is deemed to be more sensitive than long-range FCD to localize the functional organization with more alterations in local brain regions (50). Therefore, it is reasonable that more brain regions with altered functional connectivity were visualized using short-range metric. Nonetheless, through investigating the alterations of both interregional and intraregional functional interactions, this study revealed the intrinsic brain activity abnormalities mainly associated with cognitive, visual, and emotional dysfunctions in patients with TAO. Our findings might provide a new supplementary insight to the underlying neural basis of TAO disease.

This study has several limitations. First, this was a cross-sectional study. Longitudinal studies are needed to elucidate the process of functional connectivity changes and their relationships with clinical variables in the future. Second, although patients with TAO and short-term euthyroid status were recruited in this study, the potential effects of thyroid hormones on brain functional alterations cannot be fully circumvented. Future research by adding primarily patients with euthyroid TAO is warranted to verify our findings.

In conclusion, patients with TAO showed altered long- and short-range FCDs in the prefrontal cortex and parietal lobe, indicating potential cognitive, visual, and emotional dysfunctions. Our findings provided promising neuroimaging markers for assessing the psychiatric, visual, and emotional disturbances in patients with TAO.

## Data Availability Statement

The raw data supporting the conclusions of this article will be made available by the authors, without undue reservation.

## Ethics Statement

The studies involving human participants were reviewed and approved by the Ethics Committee of the First Affiliated Hospital of Nanjing Medical University. The patients/participants provided their written informed consent to participate in this study.

## Author Contributions

H-HC, X-QX, and F-YW conceptualized and designed the study. W-HJ, WC, QW, LC, and JZ performed the MR scan. W-HJ performed the MR data analyses and wrote the first draft. H-HC contributed to the diagnosis and clinical data collection. HH provided critical revisions of the draft. All authors approved the manuscript for submission. All authors contributed to the article and approved the submitted version.

## Funding

This work was supported by the National Natural Science Foundation of China (NSFC) (81801659 to HH) and Clinical Capability Promotion Project of Jiangsu Province Hospital (to X-QX).

## Conflict of Interest

The authors declare that the research was conducted in the absence of any commercial or financial relationships that could be construed as a potential conflict of interest.

## Publisher's Note

All claims expressed in this article are solely those of the authors and do not necessarily represent those of their affiliated organizations, or those of the publisher, the editors and the reviewers. Any product that may be evaluated in this article, or claim that may be made by its manufacturer, is not guaranteed or endorsed by the publisher.
